# Antimicrobial‐Resistance Genetic Markers Among Multidrug‐Resistant Enterobacteriaceae and *Acinetobacter* spp. From Vegetable Market Chains in Ethiopia

**DOI:** 10.1002/fsn3.71761

**Published:** 2026-04-12

**Authors:** Hemen Tesfaye, Adey Feleke Desta, Ahu Reis, Mine Egin, Osman Birol Ozgumus, Neslihan Akarsu, Mujib Abdulkadir, Haile Alemayehu, Tadesse Eguale, Ali Osman Kilic

**Affiliations:** ^1^ Department of Microbial Sciences and Genetics Addis Ababa University Addis Ababa Ethiopia; ^2^ Department of Medical Microbiology, Faculty of Medicine Karadeniz Technical University Trabzon Türkiye; ^3^ Department of Medical Microbiology, Faculty of Medicine Recep Tayyip Erdogan University Rize Türkiye; ^4^ Department of Biotechnology, Faculty of Science Karadeniz Technical University Trabzon Türkiye; ^5^ Aklilu Lemma Institute of Health Research Addis Ababa University Addis Ababa Ethiopia; ^6^ Ohio State Global One Health Addis Ababa Ethiopia; ^7^ Department of Medical Microbiology, Faculty of Medicine Dokuz Eylul University, Izmir Biomedicine and Genome Center Izmir Türkiye

**Keywords:** Acinetobacter spp., antimicrobial resistance, Enterobacteriaceae, resistance genes, vegetables

## Abstract

Occurrence of antimicrobial resistance (AMR) and antimicrobial resistance genes (ARGs) in Enterobacteriaceae and *Acinetobacter* spp. within agricultural environments represents a growing global health concern. Vegetables have been increasingly recognized as reservoirs of multidrug‐resistant (MDR) pathogens. The study aimed to detect ARGs in Enterobacteriaceae and *Acinetobacter* spp. from vegetable market chains and assess the clonal relationship of isolates. Antimicrobial susceptibility testing was conducted for a total of 170 isolates obtained from vegetables, irrigation water, and soil samples, and ARGs were detected by polymerase chain reaction (PCR). Conjugation assay was conducted, and genetic relatedness among bacterial isolates was assessed by ERIC‐PCR at a 70% similarity cut‐off. High resistance rate was observed to sulfamethoxazole/trimethoprim (34.0%) and cefepime (28.8%). Phenotypically, 23.5% (40/170) of isolates were extended spectrum *β*‐lactamase (ESBL) producers and from meropenem resistance isolates 60% (6/10) were carbapenemase producers. Genes encoding for broad‐spectrum *β*‐lactamases were detected in 30% (12/40) tested isolates, including those from irrigation water (50%; 3/6), vegetables (30.7%; 8/26) and soil (12.5%; 1/8) samples. Carbapenemase genes were detected in carbapenem resistant isolates from irrigation water (80%; 4/5) and vegetable (40%; 2/5) samples. The most prevalent *β*‐lactamase genes identified were *bla*CTX‐M and AmpC (*n* = 5 each), *bla*SHV (*n* = 2), and *bla*TEM (*n* = 1). Among carbapenemase genes *bla*NDM (*n* = 6) was frequently detected, and *bla*KPC and *bla*VIM in a single isolate each. A *sul1* gene was identified in 33.3% of isolates from vegetable and 15.3% of those from irrigation water. Four transconjugants were detected and successfully transferred resistance genes with conjugation frequency ranging from 1.48 × 10^−5^ to 3.3 × 10^−6^ out of 15 tested. ERIC‐PCR revealed diverse clonal lineages, although *K. pneumoniae* , *K. aerogenes* and *E. coli* isolates from vegetables and irrigation water displayed close genetic similarity (100%, 70%, and 78%), respectively. This study provided the first evidence of diverse *β*‐lactamases and carbapenemase‐producing Enterobacteriaceae in Ethiopian vegetable market chains. These findings highlight the need for continued surveillance and improved food safety measures to mitigate potential public health risks posed by antimicrobial‐resistant pathogens in the food supply chain.

## Graphical abstract

Schematic overview of the study workflow explaining isolation of lactose‐fermenting Gram‐negative bacteria from farms and supermarkets, antimicrobial susceptibility testing, detection of resistance genes by PCR, and ERIC‐PCR to evaluate genetic diversity and relatedness among isolates.

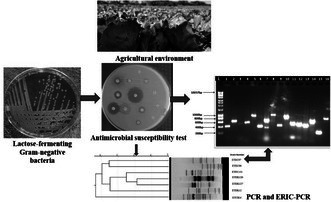

## 1 Introduction

The emergence and spread of multidrug‐resistant (MDR) Enterobacteriaceae and their genetic markers have increased significantly within both clinical as well as environmental settings, largely as a consequence of antimicrobial overuse (Pintor‐Cora et al. 2023; Richter et al. 2020). These organisms harbor antimicrobial resistance genes (ARGs) that severely limit treatment options. Resistance in MDR Enterobacteriaceae is commonly conferred by plasmid‐mediated enzymes, including ESBLs, AmpC *β*‐lactamases, and carbapenemases, which are capable of hydrolyzing third‐ and fourth‐generation cephalosporins and carbapenems (Balkhed 2014; Colosi et al. 2020; Ye et al. 2017). Among ESBLs, enzymes belonging to the family of TEM, SHV, and CTX‐M are the most frequently reported (Bush and Jacoby 2010; Coque et al. 2008; Ye et al. 2017).

Enterobacteriaceae constitute a large family of opportunistic pathogens, commonly inhabiting the intestinal tracts of humans and animals as well as diverse environmental niches (Balkhed 2014; Ye et al. 2017). Increasing evidence indicates that vegetables, irrigation water, soil, and animal manure act as important reservoirs of *β*‐lactamase‐producing Enterobacteriaceae and other opportunistic pathogens such as *Acinetobacter* spp. (Beshiru et al. 2023; Carvalheira et al. 2017; Pintor‐Cora et al. 2023; Richter et al. 2020; Son et al. 2024; Usui et al. 2019). The presence of ARGs on transferable mobile genetic elements further facilitates their dissemination (El‐Shazly et al. 2017; Usui et al. 2019). In recognition of these risks, WHO and FAO have issued warnings regarding the potential hazards of consuming fresh fruits and vegetables contaminated with resistant bacteria (FAO and WHO 2023).

The use of untreated irrigation water, contaminated soil, and animal manure in agricultural practices contributes directly to the contamination of vegetables with MDR Enterobacteriaceae and *Acinetobacter* spp. carrying resistance genetic markers (Al‐Kharousi et al. 2019; Son et al. 2024; Ye et al. 2017). Consumption of such contaminated fresh vegetables poses a risk of transmitting resistant organisms and resistance genetic markers to the human gut microbiota (Ye et al. 2017). Several studies from China, Japan, South Africa, Saudi Arabia, and Spain have reported the widespread occurrence of ESBL/AmpC‐producing Enterobacteriaceae and ARGs in vegetables and agricultural environments (Beshiru et al. 2023; Junaid et al. 2022; Pintor‐Cora et al. 2023; Richter et al. 2020; Usui et al. 2019; Ye et al. 2017).

In Ethiopia, recent studies have described the AMR profile and resistance genetic markers in *E. coli* isolated from lettuce, animal manure, and soil (Hailu et al. 2024). Additionally, phenotypic detection of *β*‐lactamase‐producing Enterobacteriaceae from market vegetables has been reported (Asfaw et al. 2023). The rapid spread of MDR bacteria in Ethiopia has been linked to the absence of regular surveillance systems and limited antibiotic stewardship programs (Kiros et al. 2021). This highlights the urgent need for more comprehensive data on the dissemination of AMR, ESBL/AmpC‐producing strains in fresh produce, as well as agricultural environments, leveraging the One Health approach.

Despite growing global evidence, knowledge regarding the occurrence and type of ARGs in circulating MDR Enterobacteriaceae and *Acinetobacter* spp. in Ethiopian vegetable market chains remains limited. Therefore, this study aimed to identify and characterize Enterobacteriaceae and *Acinetobacter* spp. in commonly consumed vegetables (from farms and supermarkets), irrigation water, and soil samples collected from farms in Addis Ababa city and the Oromia region, one of the country's major vegetable‐producing areas.

## 2 Methods

### 2.1 Sampling Sites and Sample Collection

Samples were collected from two GLOBAL G.A.P certified farms, and four smallholder farms in Oromia region (Holeta, Bishoftu, Koka, Sheger city), and six supermarkets in Addis Ababa, Ethiopia. With the exception of one Global‐GAP certified farm, all farms used untreated river water for irrigation and holding dams. In total 408 samples were collected, including 24 irrigation water, 48 soil and 336 vegetable samples.

Fresh vegetable samples (lettuce, carrot, cabbage, and tomato (150 g each)) were collected in sterile zippered plastic bags and transported to the Microbiology Laboratory, College of Natural and Computational Sciences, Addis Ababa University for analysis. Soil samples were collected from three random sites per field from three plots (0–20 cm depth), each 6 m apart. Five subsamples from each plot were pooled to obtain one composite soil sample of 250 g. Water was sampled at the inlet point prior to storing in a holding dam at the point of irrigation using 500 mL sterile bottles. All aseptically collected samples were transported to the laboratory using an ice box containing an ice pack.

### 2.2 Isolation and Identification of Enterobacteriaceae and *Acinetobacter* spp.

A150 g portion of each vegetable samples were placed in 150 mL of buffered peptone water (BPW) at a 1:1 w/v ratio, while lettuce (25 g each) was placed in 250 mL BPW at a 1:10 w/v ratio and incubated at 37°C for 18–24 h for the isolation of Enterobacteriaceae and *Acinetobacter* spp. (Richter et al. 2019). Similarly, 10 g of each composite sample were placed into 90 mL of sterile BPW and homogenized thoroughly to form a suspension. The enrichment samples were incubated at 37°C for 18–24 h for the detection of presumptive Enterobacteriaceae and *Acinetobacter* spp. (Zekar et al. 2017). Similarly, 1 mL of water sample was pre‐enriched in nine milliliters of BPW and incubated at 37°C for 3 to 4 h before inoculation on selective media (Richter et al. 2020).

Enriched samples were streaked onto MacConkey agar and Eosin Methylene Blue agar and incubated overnight at 37°C. Presumptive isolates were purified and initially identified based on IMViC biochemical tests for Enterobacteriaceae, and by colony morphology and oxidase test for *Acinetobacter* spp. Final confirmation of bacterial species was performed using matrix‐assisted laser desorption/ionization time‐of‐flight mass spectrometry (MALDI‐TOF MS; Bruker, Bremen, Germany) (Schaumann et al. 2013).

### 2.3 Antimicrobial Susceptibility Testing

Antimicrobial susceptibility testing was conducted using the Kirby‐Bauer disk diffusion method following Clinical and Laboratory Standards Institute's (CLSI) guidelines (CLSI 2024). Sixteen antimicrobials were tested: piperacillin/tazobactam (100/10 μg), amoxicillin/clavulanic acid (20/10 μg), gentamicin (10 μg), amikacin (30 μg), tetracycline (30 μg), ampicillin (10 μg), cefazolin (30 μg), cefoxitin (30 μg), ceftazidime (30 μg), ceftriaxone (30 μg), cefepime (30 μg), meropenem (10 μg), ciprofloxacin (5 μg), sulfamethoxazole/trimethoprim (23.75/1.25 μg), Cefotaxime (30 μg), and chloramphenicol (30 μg) (Sensi Discs, Becton, Dickinson and Company, Loveton, USA). *E. coli* ATCC 25922 was used as a quality control strain. The inhibition zones were measured using caliper and interpreted as resistant, intermediate, or susceptible as per CLSI guidelines (CLSI 2024).

### 2.4 Detection of Extended‐Spectrum *β*‐Lactamase Production

Isolates exhibiting intermediate or full resistance to 2^nd^ or 3^rd^ generation cephalosporins were screened for ESBL production using double‐disc synergy test (DDST) (Drieux et al. 2008). Amoxicillin‐clavulanate (20/10 μg) and ceftazidime (30 μg) discs were kept 20 mm apart. Synergy, indicated by expansion of the inhibition zone of ceftazidime towards amoxicillin/clavulanate, was interpreted as positive for ESBL production (Kaur et al. 2013). All isolates exhibiting the ESBL phenotypes were subsequently tested by PCR for the presence of different *β*‐lactamase encoding genes like *bla*TEM, *bla*SHV, *bla*CTX‐M, and AmpC‐type *β*‐lactamase genes.

### 2.5 Detection of Carbapenemase Production

Carbapenemase activity was assessed using the modified carbapenem inactivation assay (mCIM), following CLSI guidelines (CLSI 2024) for those Enterobacteriaceae species resistant to meropenem. Briefly, meropenem (10 μg) discs were immersed in test tubes containing bacterial suspensions prepared in Mueller–Hinton broth and incubated for 4 h at 37°C. The discs were then placed on Mueller–Hinton agar plates swabbed with *E. coli* ATCC 25922 and incubated at 37°C for 18–24 h. Carbapenemase production was considered positive if the inhibition zone measured 6–15 mm, or if colonies were present within a 16–18 mm zone.

### 2.6 Identification of Antimicrobial Resistance Genes

Single colonies of Enterobacteriaceae and *Acinetobacter* spp. isolates were cultured overnight in 10 mL Luria–Bertani (LB) broth under shaking at 200 rpm. Cells were harvested by centrifugation (12,500× *g*, 10 min), and genomic DNA was extracted using the phenol–chloroform method (Chen and Tsong 1993). Isolates showing phenotypic resistance to tetracycline, ciprofloxacin, sulfonamides, meropenem, ampicillin, or cefotaxime were screened for 16 ARGs by conventional PCR including genes encoding for tetracycline (*tetA* and *tetB*), quinolone (*qnrA*, *qnrB*, and *qnrS*), sulfonamides (*sul1* and *sul2*), *β*‐lactamases (*bla*TEM, *bla*SHV, *bla*CTX‐M, and AmpC). PCR products were visualized by agarose gel electrophoresis. Primer sequences and PCR conditions used, and amplicon size of the PCR products are shown in Table S1.

### 2.7 Genomic Fingerprinting of Enterobacteriaceae and *Acinetobacter* spp. by ERIC‐PCR

Selected Enterobacteriaceae and *Acinetobacter* spp. *β*‐lactamase and carbapenemase producers were subjected to repetitive sequence‐based PCR (ERIC‐PCR) to generate genomic fingerprints from all sampling sources. PCR reactions were carried out at a total volume of 25 μL, containing 2X GoTaq Green Master Mix (Promega Corp. USA), 0.2 μM of each primer, and 50 ng (2 μL) of template DNA. The primers used were ERIC1R (5′‐ATG TAA GCT CCT GGG GAT TCA C‐3′) and ERIC2 (5′‐AAG TAA GTG ACT GGG GTG AGC G‐3′) (Sekhar et al. 2017). Amplification conditions and cycling parameters followed the protocol described by Sekhar et al. (2017). PCR products were separated on 1.5% agarose gel and visualized under UV illumination. Clonal relatedness was assessed using ERIC‐PCR with a 70% similarity threshold applied for clustering.

### 2.8 Conjugation Assay and Plasmid Isolation

Conjugation assay was performed using the broth mating method (Ozgumus et al. 2008). *E. coli* J53‐2 (ampicillin sensitive and rifampicin resistant) was used as the recipient strain. A total of 15 donor isolates, either positive for *β*‐lactamase production and/or carbapenemase enzyme, were selected. Transconjugants were selected on LB agar containing rifampicin (300 μg/mL) and ampicillin (200 μg/mL). Conjugation frequency was calculated as the number of transconjugants per donor cell.

Plasmid DNA was isolated from both donor and transconjugants using the alkaline lysis method (Manniatis et al. 1982). The size of the plasmids was estimated by comparing plasmid marker ( *E. coli* V517) with each purified sample (transconjugant and donor cell) on 1% agarose gel at 70 V for 2 h. Plasmid DNA from transconjugants was subsequently used as a template for PCR amplification of resistance genes. Donor isolates carrying AmpC, *bla*TEM, *bla*SHV, *bla*CTX‐M, and carbapenemase genes served as positive controls. Transconjugants were further subjected to antimicrobial susceptibility testing, and their phenotypic resistance profiles were compared with those of donor strains to confirm successful transfer.

### 2.9 Statistical Analysis

Data was compiled in Microsoft Excel and analyzed using descriptive statistics, including percentage distributions. Antimicrobial susceptibility profiles and associated resistance genes across sample types were visualized as a heatmap using Python software (version 3.11). ERIC PCR fingerprint data were analyzed with GelJ software (version 2.0). Dendrograms were constructed using the Jaccard similarity coefficient and the unweighted pair group method with arithmetic mean (UPGMA).

## 3 Results

### 3.1 Antimicrobial Susceptibility Profiles

Susceptibility of the 170 bacterial isolates to different antimicrobials varied by species (Table 1). *E. coli* (15/45; 33.3%) exhibited high rate of resistance to ampicillin and amoxicillin/clavulanic acid, whereas *Klebsiella aerogenes* (9/9; 100%) showed high resistance rate to cefoxitin. The highest rate of resistance was recorded for *K. oxytoca* to piperacillin/tazobactam (2/3; 66.7%). Notable high resistance rates included *Citrobacter braakii* to ceftazidime (3/5; 60%), *E. coli* to sulfamethoxazole/trimethoprim (28/45; 62.2%), *K. pneumoniae* to meropenem (5/32; 15.6%), ciprofloxacin (12/32; 37.5%), and *Citrobacter freundii* to tetracycline (10/16; 62.5%). Overall, resistance to sulfamethoxazole/trimethoprim was highest across all isolates from different species (58/170; 34%), followed by cefepime (49/170; 28.8%), cefoxitin and ceftazidime (44/170; 25.8%).

**TABLE 1:** Antimicrobial resistance profile of the Enterobacteriaceae and *Acinetobacter* spp. isolated from vegetables, irrigation water and soil samples.

Species of bacteria	Number and (%) resistant to antimicrobial tested															
	AMP	AMC	PTZ	GM	AK	TE	CZ	FOX	CTX	CAZ	CRO	FEP	MEM	CIP	SXT	C
*A. baumannii* (5)	NA	NA	1 (20)	0	2 (40)	0	0	1 (20)	1 (20)	1 (20)	1 (20)	0	0	0	3 (60)	NA
*A. pittii* (12)	NA	NA	3 (25)	4 (33.3)	3 (25)	3 (25)	5 (41.6)	1 (8.3)	2 (16.6)	4 (33.3)	4 (33.3)	3 (25)	0	2 (16.6)	2 (16.6)	NA
*A. junii* (1)	NA	NA	0	1 (100)	1 (100)	0	0	0	0	0	1 (100)	0	0	0	0	NA
*C. braakii* (5)	NA	NA	0	1 (20)	0	1 (20)	NA	3 (60)	1 (20)	3 (60)	0	0	0	1 (20)	0	0
*C. freundii* (16)	NA	NA	3 (19)	3 (19)	0	10 (62.5)	NA	NA	3 (19)	4 (25)	7 (44)	5 (31.3)	0	4 (25)	4 (25)	2 (12.5)
*E. asburiae* (17)	NA	NA	3 (17.6)	3 (17.6)	0	4 (23.5)	NA	NA	4 (23.5)	5 (29.4)	2 (11.7)	3 (17.6)	1 (5.8)	4 (23.5)	2 (11.7)	2 (11.7)
*E. bugandensis* (4)	NA	NA	0	0	0	0	NA	NA	1 (25)	2 (50)	1 (25)	2 (50)	0	0	0	0
*E. cloacae* (5)	NA	NA	2 (40)	2 (40)	0	0	NA	NA	0	0	1 (20)	2 (40)	0	0	1 (20)	0
*E. coli* (45)	15 (33.3)	15 (33.3)	15 (33.3)	10 (22.2)	1 (2.2)	12 (26.7)	4 (9)	7 (15.6)	7 (15.6)	12 (26.7)	15 (33.3)	22 (48.9)	4 (9)	9 (20)	28 (62.2)	4 (8.9)
*E. kobei* (4)	NA	NA	0	0	0	0	NA	NA	0	0	0	0	0	0	1 (25)	0
*K. aerogenes* (9)	NA	NA	3 (33.3)	1 (11.1)	0	2 (22.2)	NA	9 (100)	3 (33.3)	4 (44.4)	1 (11.1)	3 (33.3)	0	3 (33.3)	5 (55.5)	0
*K. oxytoca* (3)	NA	2 (66.7)	2 (66.7)	2 (66.7)	0	0	2 (66.7)	1 (33.3)	1 (33.3)	1 (33.3)	1 (33.3)	2 (66.7)	0	0	0	0
*K. pneumoniae* (32)	NA	5 (15.6)	7 (21.9)	3 (9.4)	1 (3.1)	4 (12.5)	2 (6.3)	15 (46.9)	5 (15.6)	5 (15.6)	6 (18.8)	4 (12.5)	5 (15.6)	12 (37.5)	11 (34.4)	3 (9.4)
*K. variicola* (12)	NA	5 (41.6)	2 (16.6)	5 (41.6)	2 (16.6)	4 (33.3)	5 (41.6)	7 (58.3)	1 (8.3)	3 (25)	5 (41.6)	3 (25)	0	3 (25)	1 (8.3)	2 (16.6)
Total (170)	15 (8.8)	27 (15.8)	41 (24)	35 (20.5)	10 (5.8)	40 (23.5)	18 (10.5)	44 (25.8)	29 (17)	44 (25.8)	45 (26.4)	49 (28.8)	10 (5.8)	38 (22.3)	58 (34)	13 (7.6)

Abbreviations: AK, Amikacin; AMC, Amoxicillin/clavulanic acid; AMP, Ampicillin; C, Chloramphenicol; CAZ, Ceftazidime; CIP, Ciprofloxacin; CRO, Ceftriaxone; CTX, Cefotaxime; CZ, Cefazolin; FEP, Cefepime; FOX, Cefoxitin; GM, Gentamicin; MEM, Meropenem; NA, not applicable; PTZ, Piperacillin/Tazobactam; SXT, Sulfamethoxazole/Trimethoprim; TE, Tetracycline.

### 3.2 ESBL and Carbapenemases Production

The ESBL phenotype was detected in 23.5% (40/170) of the Enterobacteriaceae (*n* = 38) and *Acinetobacter* spp. (*n* = 2) isolates that exhibited intermediate or full resistance to third‐generation cephalosporins. Detection of ESBLs was highest among isolates obtained from soil 29.6% (8/27) followed by those from irrigation water 25% (6/24) and those from vegetables 21.8% (26/119), although this difference was not statistically significant (*p* = 0.67). The distribution of ESBL producing bacterial species is summarized in Table 2.

**TABLE 2:** Rate of occurrence of ESBL production among Enterobacteriaceae and *Acinetobacter* spp. using a double disc synergy test.

Bacteria species	DDST result	No. positive	%Positive	Sampling type for positive isolates		
	No. tested			Vegetables (*n* = 119)	Water (*n* = 24)	Soil (*n* = 27)
*A. baumannii*	5	0	0	—	—	—
*A. pittii*	7	2	28.5	2	—	—
*C. braakii*	4	1	25	1	—	—
*C. freundii*	13	0	0	0	—	0
*E. coli*	39	12	30.7	9	2	1
*E. asburiae*	27	5	18.5	1	2	2
*E. bugandensis*	7	2	14.2	1	—	1
*E. cloacae*	8	3	37.5	2	—	1
*E. kobei*	4	3	75	2	—	1
*K. aerogenes*	9	2	22.2	2	—	—
*K. oxytoca*	3	0	0	—	—	—
*K. pneumoniae*	32	8	25	4	2	2
*K. variicola*	12	2	16.6	2	—	—
Total	170	40 (23.5)		26 (21.8)	6 (25)	8 (29.6)

Abbreviation: DDST, double disc synergy test.

Phenotypic carbapenemase production was confirmed using the mCIM test in 60% (6/10) of Enterobacteriaceae isolates resistant to meropenem. Among these carbapenemase‐producing isolates, the majority were from irrigation water (3 *K. pneumoniae* and 1 *E. coli* ), whereas 2 *K. pneumoniae* isolates were from vegetables. None of the isolates from soil were phenotypically positive for carbapenemase production (Table 3).

**TABLE 3:** Distribution of carbapenemase producing isolates among vegetables, irrigation water and soil samples.

Bacteria species	mCIM	Sampling type for positive isolates		
	No. positive %	Vegetables (*n* = 5)	Water (*n* = 5)	Soil (*n* = 0)
*E. coli*	1 (25)	—	1	—
*E. asburiae*	0 (0)	—	—	—
*K. pneumoniae*	5 (100)	2	3	—
Total	6 (60)	2 (40)	4 (80)	0 (0)

Abbreviation: mCIM, modified carbapenem inactivation assay.

### 3.3 Genetic Markers Associated With Antimicrobial Resistance Among Enterobacteriaceae and *Acinetobacter* spp.

**FIGURE 1:**
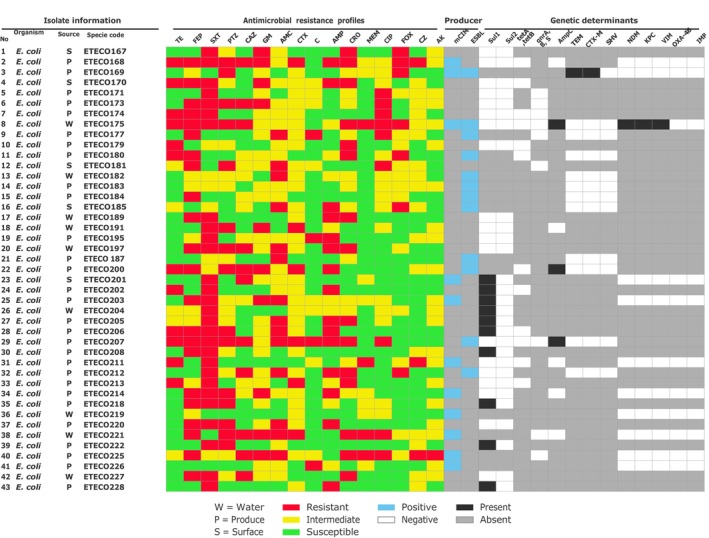
Heatmap of phenotypic resistance pattern and their genetic determinant of selected resistance phenotype of *E. coli* . AK, Amikacine; AMC, Amoxicillin/clavulanic acid; AMP, Ampicillin; C, Chloramphenicol; CAZ, Ceftazidime; CIP, Ciprofloxacin; CRO, Ceftriaxone; CTX; Cefotaxime; CZ; Cefazolin; FEP, Cefepime; FOX, Cefoxitin; GM, Gentamicin; MEM, Meropenem; PTZ, Piperacillin/Tazobactam; SXT, Sulfamethoxazole/Trimethoprim; TE, Tetracycline.

**FIGURE 2:**
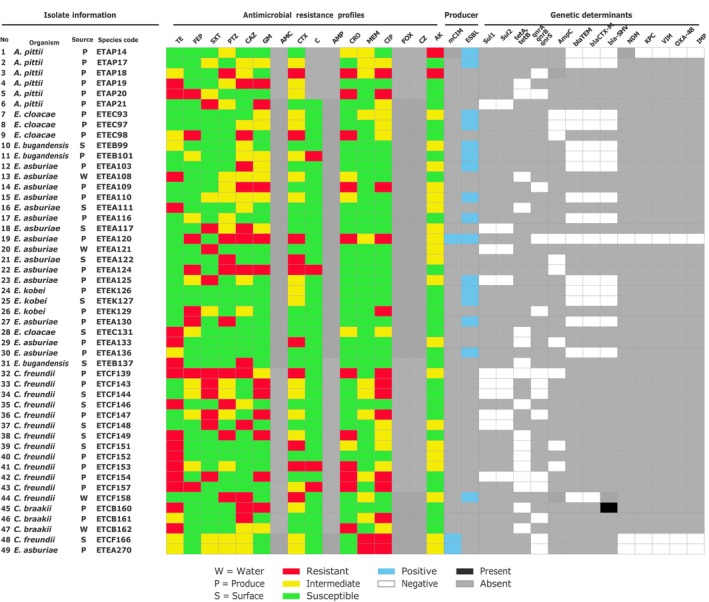
Heatmap of phenotypic resistance pattern and their genetic determinant of selected resistance phenotype of *A. baumannii* , *A. pittii* , *C. braakii* , *C. freundii* , *E. asburiae* , *E. bugandensis*, *E. cloacae* , *E. kobei* . AK, Amikacine; AMC, Amoxicillin/clavulanic acid; AMP, Ampicillin; C, Chloramphenicol; CAZ, Ceftazidime; CIP, Ciprofloxacin; CRO, Ceftriaxone; CTX, Cefotaxime; CZ, Cefazolin; FEP, Cefepime; FOX, Cefoxitin; GM, Gentamicin; MEM, Meropenem; PTZ, Piperacillin/Tazobactam; SXT, Sulfamethoxazole/Trimethoprim; TE, Tetracycline.

Phenotypically resistant Enterobacteriaceae and *Acinetobacter* spp. were screened for their respective genetic markers. Overall, *β*‐lactamase genes were detected in 30% (12/40) tested isolates, including those from vegetables (30.7%; 8/26), irrigation water (50%; 3/6), and soil (12.5%; 1/8). These isolates mainly comprise *E. coli* (*n* = 4), *K. pneumoniae* (*n* = 4), *K. variicola* (*n* = 2), *K. aerogenes* (*n* = 1), and *Citrobacter braakii* (*n* = 1). Among them, 58.3% (7/12) carried *bla*CTX‐M, *bla*TEM, and *bla*SHV, while 12.5% (5/12) harbored AmpC *β*‐lactamase genes. The most prevalent *β*‐lactamase genes identified were *bla*CTX‐M and AmpC (each *n* = 5), followed by *bla*SHV (*n* = 2) and *bla*TEM (*n* = 1). Carbapenemase‐associated genes were detected in 60% (6/10) tested isolates, specifically in those from irrigation water (80%; 4/5) and vegetables (40%; 2/5). *E. coli* from all sampling sources displayed diverse resistance genes. Vegetable‐derived isolates carried *bla*TEM and *bla*CTX‐M, whereas one isolate from irrigation water harbored multiple resistance determinants, including AmpC and carbapenemase genes (KPC, NDM, and VIM). Another vegetable‐derived *E. coli* isolate carried AmpC (Figure 1). *C. braakii* isolates detected from vegetables carried *bla*SHV genes (Figure 2).

Sulfonamide resistance genes, primarily *sul1*, were found in 22.5% (14/62) of SXT resistant isolates, including vegetables (33.3%; 12/36) and irrigation water (15.3%; 2/13), whereas *sul2* was not detected in any of the isolates. Majority of the *sul1* gene was detected from *E. coli* isolates (*n* = 10), most of them from isolates originating from vegetables. None of the isolates tested carried OXA‐48 like, IMP, *tetA*, *tetB*, *qnrA*, *qnrB*, and *qnrS* genes.

**FIGURE 3:**
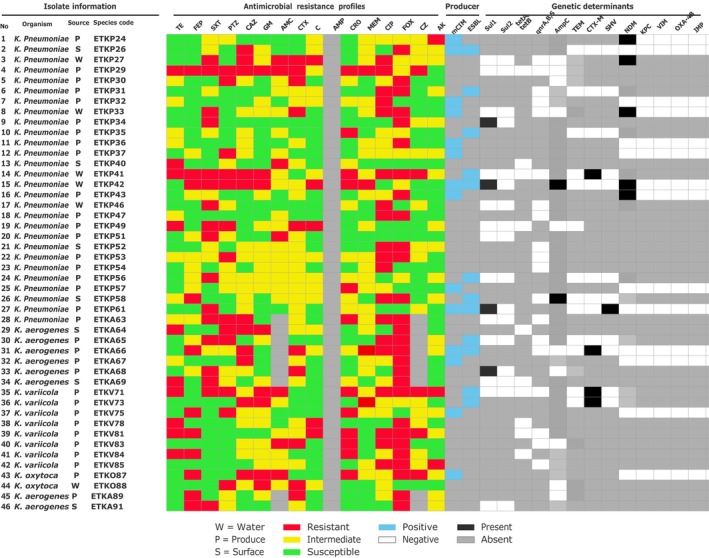
Heatmap of phenotypic resistance pattern and their genetic determinant of selected resistance phenotype of *K. pneumoniae* , *K. aerogenes* , *K. variicola* , *K. oxytoca* . AK, Amikacine; AMC, Amoxicillin/clavulanic acid; AMP, Ampicillin; C, Chloramphenicol; CAZ, Ceftazidime; CIP, Ciprofloxacin; CRO, Ceftriaxone; CTX, Cefotaxime; CZ; Cefazolin; FEP, Cefepime; FOX, Cefoxitin; GM, Gentamicin; MEM, Meropenem; PTZ, Piperacillin/Tazobactam; SXT, Sulfamethoxazole/Trimethoprim; TE, Tetracycline.

Our results showed that the *K. pneumoniae* detected from vegetables carried the NDM, *sul1* gene, while isolates from irrigation water harbored *bla*CTX‐M, NDM, and those from soil carried AmpC resistance genes. Notably, a single *K. pneumoniae* isolated from irrigation water carried three resistance determinants, including *sul1*, AmpC, and NDM. In addition, one vegetable‐derived isolate carried both *sul1* and *bla*SHV genes. Among other species, two *K. aerogenes* from vegetables were positive for *bla*CTX‐M and *sul1* genes, while two *K. variicola* isolates from vegetables carried *bla*CTX‐M genes (Figure 3).

### 3.4 Genomic Fingerprinting of Enterobacteriaceae and *Acinetobacter* spp. by ERIC‐PCR

**FIGURE 4:**
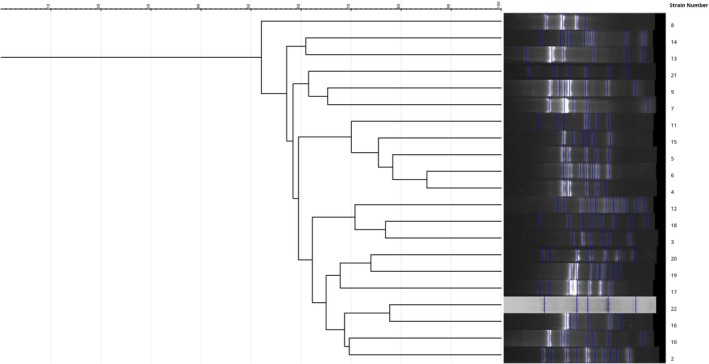
Dendrogram showing fingerprinting profiles of *E. coli* phenotypically ESBL/AmpC and carbapenems producer strains isolated from Farms and markets (vegetable, soil and irrigation water samples). The profiles were obtained with ERIC‐PCR.

*E. coli* isolates from vegetables (ETECO 200) and soil (ETECO 201) collected from the same farm shared 72% similarity in DNA fingerprinting patterns (Figure 4; strain number correspond to Table S2). Likewise, *E. coli* isolates from vegetables (ETECO 212) and irrigation water (ETECO 219) obtained from the same farm showed a high degree of relatedness (78% similarity). In addition, *E. coli* isolates from market vegetables (ETECO 211) and farm vegetables (ETECO 177) exhibited identical patterns. Although collected from different farms, two *E. coli* isolates, one from vegetable (ETECO 183) and the other from soil (ETECO 185), were closely related. Despite these instances of clonal similarity, most *E. coli* isolates from vegetables, soil, and irrigation water exhibited diverse ERIC‐PCR profiles, suggesting heterogeneity of strains circulating within farms and markets.

A total of 15 *K. pneumoniae* isolates were screened and isolates from vegetables (ETKP 35 and ETKP37) obtained from different farms exhibited 100% clonal similarity values. Similarly, ETKP57 and ETKP61 isolated from vegetables on the same farm showed a closely related banding pattern. Similarity at 76% was observed in *K. pneumoniae* isolates from two different markets (ETKP43 and ETKP56). On the other hand, *K. pneumoniae* isolated from irrigation water (ETKP33) and vegetables (ETKP36) from different farms showed 72% clonal similarity. However, *K. pneumoniae* isolated from vegetables (ETKP24), soil (ETKP26, ETKP58), and irrigation water (ETKP41, ETKP42) from different farms showed more diverse fingerprint patterns (Figure S1; strain numbers correspond to Table S3).

The three *K. variicola* isolated from vegetables were not clonally related. Two *E. asburiae* isolated from vegetables (ETEA103) and irrigation water (ETEA108) on two different farms, showed identical patterns, whereas the remaining isolates showed different patterns (Figure S2; strain numbers correspond to Table S4). *K. aerogenes* (ETKA66 and ETKA67) isolated from vegetables on same farms exhibited related DNA fingerprinting patterns. But a different band pattern was observed in a single *K. aerogenes* (ETKA65) isolated from the market. *A. pittii* (ETAP6) isolated from a farm and (ETAP8, ETAP17, and ETAP18) market vegetable sample showed diverse clonal relationships. Likewise, *A. baumannii* isolated from irrigation water (ETAB2 and ETAB3), and soil (ETAB5) were genetically distinct variants. Moreover, all *E. bugandensis* (ETEB99, ETEB101, and ETEB137) species isolated from soil and market showed more diverse clonal relatedness (Figure S3; strain numbers correspond to Table S5). *E. kobei* isolated from vegetables (ETEK 126) and soils (ETEK127) from the same farm showed genetic relatedness at 83% similarity (Figure S4; strain numbers correspond to Table S6).

### 3.5 Transfer of Phenotypic Resistance and *β*‐Lactamase and Carbapenemase Encoding Genes

Out of 15 isolates for which conjugation assay was conducted, 4 of them were able to transfer genetic mobile elements to the transconjugants. All transconjugants showed similar resistance profile with the donor strain (Table 4). The only exception is strain of *E. coli* (ETECO160) which exhibited transfer of resistance profile except for ceftazidime to the transconjugant. The result of this experiment also showed that a total of *β*‐lactamase and carbapenemase genes producing isolates were able to transfer their *β*‐lactamase and carbapenemase genes to recipient ( *E. coli* J53‐2) by conjugation at the range of 1.48 × 10^−5^ to 3.3 × 10^−6^ conjugation frequency (Table 4). The gel electrophoresis of donor strains and transconjugants yielded a single band with the same plasmid size around 50 kb (Figure S5).

**TABLE 4:** Conjugation frequency and resistance profile of transconjugants.

Transconjugant	Phenotype resistance profile		*bla* genes	Frequency of transfer (cfu/recipient)
	Donor	Transconjugants		
TXG42	CRO, AMP, CAZ, AMC, FEP, MEM, PTZ, FOX, SXT, GM, C	CRO, AMP, CAZ, AMC, FEP, MEM, PTZ, FOX, SXT, GM, C	NDM, AmpC	3.3 × 10^−6^
TXG160	PTZ, FOX, CAZ, CTX	PTZ, FOX, CTX	SHV	2.13 × 10^−5^
TXG169	PTZ, FOX, CTX	PTZ, FOX, CTX	CTX‐M and TEM	1.4 × 10^−5^
TXG200	CAZ, AMP, AMC, TE, SXT, FEP, PTZ, CTX	CAZ, AMP, AMC	AmpC	2.2 × 10^−4^

Abbreviations: AMC, Amoxicillin+clavulanic acid; AMP, Ampicillin; C, Chloramphenicol; CAZ, Ceftazidime; CRO, Ceftriaxone; CTX, Cefotaxime; FEP, Cefepime; FOX, Cefoxitin; GM, Gentamicin; MEM, Meropenem; PTZ, Piperacillin/Tazobactam; SXT, Sulfamethoxazole/Trimethoprim; TE, Tetracycline.

## 4 Discussion

The present investigation provides insight into antimicrobial resistance profile and genetic markers associated with *β*‐lactamase and carbapenemase production and other resistance genes among Enterobacteriaceae and *Acinetobacter* spp. within the production environment and vegetable market chains. The majority of *E. coli* isolates in this study were resistant to sulfamethoxazole/trimethoprim (62.2%), similar to the previous report (56%) from Addis Ababa (Hailu et al. 2024), whereas resistance to ampicillin (33.3%) and amoxicillin/clavulanic acid (33.33%) is relatively low, contrary to a previous report, which was 52% and 43%, respectively (Hailu et al. 2024). The observed difference in the resistance profile of *E. coli* could be associated with variation in agricultural practices and environmental factors. The previous study was conducted in Addis Ababa where farmers use animal manure as fertilizer, which could be the source of exposure to antimicrobial resistant organisms and genetic markers from animals treated with antimicrobial agents, increasing selection pressure.

Resistance to meropenem was observed in 6.5% (10/152) of Enterobacteriaceae strains in the current study, closely related to the previous study which reported 13.7% among gram‐negative organisms isolated from vegetables sold at Debre Berhan town, Ethiopia (Asfaw et al. 2023). On the other hand, no resistance to meropenem was reported from a similar previous study in Addis Ababa, Ethiopia (Hailu et al. 2024). In our study, the highest rate of resistance was recorded across all isolates in vegetables, irrigation water, and soil samples to sulfamethoxazole/trimethoprim 34% (58/170) and cefepime 28.8% (49/170). This shows a potential route for the dissemination of bacterial strains and associated resistance genes from the agricultural environment to humans through the food chain. Moreover, this shows how the agricultural environment may serve as a reservoir for multidrug resistance to opportunistic human pathogens in vegetable production.

Detection of *β*‐lactamase encoding gene producers in vegetables, irrigation water and soil at a rate of over 21% in the current study is relatively comparable with a previous report from small holder vegetable farms from South Africa (Viviers et al. 2025), among ESBL producing ESKAPE‐E pathogens isolated from vegetables (54%), soil (31%) and irrigation water (15%). In contrast, 80.5% of vegetables were found to be phenotypically positive for ESBL/AmpC producing Enterobacteriaceae from vegetables and salads in South Africa (Beshiru et al. 2023), and 11.6% from irrigation water in China (Ye et al. 2017). Overall rate of carbapenem resistance (5.8%) and carbapenemase production among Enterobacteriaceae (3.5%) was recorded in the current study. This finding is lower than the 16.3% carbapenem resistance from a previous report in isolates obtained from irrigation water in China (Guo et al. 2023) and (30%) reported from fresh produces and farms in Egypt (Elshafiee et al. 2022).

Carbapenemase producing Enterobacteriaceae and *Acinetobacter* spp. were more common among isolates from irrigation water compared to those obtained from vegetable samples, whereas none were detected among those isolated from soil samples. This finding suggests that contaminated irrigation water may be a possible source of environmental contamination with carbapenem‐resistant organisms, and vegetables may also become contaminated through human contact (Soler et al. 2025).

Isolates obtained from irrigation water had the highest rate of ARGs 37.5% (9 of 24) (*sul1*, *β*‐lactamase genes and carbapenemase genes) compared to those from vegetables 6.54% (22 of 336) and those obtained from soil 2% (1 of 48). A plausible justification for this observation could be irrigation water as a reservoir of resistance genes that can spread to farm environment and vegetables. Irrigation water was reported as the highest reservoir of potential human pathogens and ARGs in previous studies (Al‐Kharousi et al. 2019; Richter et al. 2020). Surprisingly, overall, a limited number of Enterobacteriaceae were positive for tested ARGs in our study. *β*‐lactamase genes (*bla*CTX‐M, *bla*TEM and *bla*SHV) 17.5% (7 of 40) were detected; however, ESBL variants of *bla*TEM/*bla*SHV were not confirmed by sequencing from our isolates. Mainly, 23% (6/26) of vegetables, and 16.6% (1/6) of irrigation water, which is lower than 64.42%, similar studies in South Africa (Richter et al. 2020) and 37% reported in Central Chile (Diaz‐Gavidia et al. 2021). Mostly, ESBL prevalence varies among countries. Such variability in the detection of ARGs may be due to the presence of multiple resistance mechanisms encoding resistance to various antimicrobials, and the fact that our study was not exhaustive enough to cover all target genetic markers. In addition, the epidemiology of resistance genes may vary due to differences in antimicrobial use between clinical and livestock settings.

The potential pathogenic *bla*CTX‐M, *bla*TEM, and *bla*SHV producing isolates were detected from our sample, corresponding to previous findings (Chelaghma et al. 2022; Giri et al. 2021; Ye et al. 2017). An *E. coli* isolated from vegetables harbored two ESBL resistance genes: *bla*TEM and *bla*CTX‐M. Along with our study, *E. coli* detected from the farm environment has also been reported to carry *bla*CTX‐M and other ESBL genes (Hartmann et al. 2012), while *bla*CTX‐M was also detected from *K. pneumoniae* in irrigation water, which is similar to previous findings by Richter et al. (2019). Moreover, two *K. variicola* and a single *K. aerogenes* are isolated from vegetable samples carrying *bla*CTX‐M genes. These genes are widely distributed globally and frequently reported in various studies in clinical and agricultural settings (Colosi et al. 2020; Diaz‐Gavidia et al. 2021; Elraghya et al. 2016). In Ethiopia, ESBLs are the most frequently utilized antimicrobial groups (Gutema 2023), and *β*‐lactamase genes (CTX‐M, TEM, SHV) are highly reported genes from clinical, environmental, and agricultural isolates (Hailu et al. 2024; Worku et al. 2025).

To the best of our knowledge, this is the first report on the Enterobacteriaceae family and *Acinetobacter* spp. isolates harboring AmpC and carbapenemase resistance genes in Ethiopia, particularly from samples collected from the agricultural environment. AmpC resistance gene was detected in 12.5% (5 of 40) *E. coli* and *K. pneumoniae* isolates from vegetables, soil, and irrigation water. These two species were extensively investigated globally and commonly isolated from the environment and clinical settings. Similar studies reported detection of AmpC producing *E. coli* and *K. pneumoniae* from sources of various vegetables (Richter et al. 2020), and soil samples (Viviers et al. 2025; Ye et al. 2017). Most of the *β*‐lactamase genes like AmpC in selected gram‐negative species are located on plasmids mediated with the capability of transferring resistance genes to other bacteria by horizontal gene transfer. The detection of these plasmid‐mediated genes in vegetables poses a serious public health concern.

Detection of carbapenem resistance genes dominated by NDM in *E. coli* and *K. pneumoniae* isolated from irrigation water and vegetables provides new insights into the agricultural settings as a potential reservoir of carbapenem resistance organisms in Ethiopia. Similarly, Enterobacteriaceae exhibiting *bla*NDM‐5 were reported from vegetables and irrigation water in China (Zhao et al. 2021), and in Egypt, *K. pneumoniae* carrying *bla*NDM was reported from river irrigation water (Elsherbeny et al. 2024). The potential reason for a few isolates phenotypically resistant to carbapenems but missing known carbapenem resistance genetic markers could be due to other resistance mechanisms. A previous study showed ertapenem resistance in *E. coli* and *K. pneumoniae* isolates without carbapenemase genes was due to a combination of ESBLs and AmpC overproduction and loss of porin (Johnning et al. 2018). To come up with a clear picture of the genetic markers associated with carbapenem resistance, conducting whole genome sequencing (WGS) is recommended.

Resistance gene encoding sulfonamide antimicrobials (*sul1*) was detected in 22.5% (14/62) of isolates in the current study. Similarly, *sul1* gene was reported in other studies among isolates obtained from irrigation water (Amato et al. 2021; Iwu et al. 2020; Shamsizadeh et al. 2021). The possible reason for high rate of resistance to sulfonamide and wide occurrence of *sul1* gene is that the sulfonamide group of antimicrobials has been widely used to treat bacterial infections in animals in Ethiopia (Messele et al. 2017). In this study, *sul2*, *tet(A)*, *tet(B)*, *qnrA*, *qnrB* and *qnrS* resistance genes were not detected. Similar study in Ethiopia also reported the same result (Hailu et al. 2024), but opposite to a study conducted in south Africa (Iwu et al. 2020) where they have detected these resistance genes. The possible reason for the absence of some genes we tested in our isolates could be due to other genes like *tet(C)*, *tet(O)*, *tet(M)*, *sul3*, *qnrD* and aac(6′)‐Ib‐cr contributing to the observed phenotypic resistance where we did not test for these genes.

In current study, plasmid mediated resistance determinants were successfully transferred via broth mating assays for 4 out of 15 (26.6%) tested isolates. These results are consistent with earlier research showing that the horizontal transfer of MDR through plasmids among Enterobacteriaceae (Ozgumus et al. 2008; Hartmann et al. 2012; Saliu et al. 2020). However, majority of isolates were not able to transfer their resistance genes to the recipient strains probably due to the fact that plasmids carried by these isolates may not have F‐pilus which enable the efficient horizontal transfer (Zatyka and Thomas 1998).

According to ERIC‐PCR, *E. coli* isolates from vegetable and irrigation water showed a high degree of genetic relatedness at 78% similarity values on the same farms, and 72% similarity on market of respective chains, which is similar to results observed in previous studies (Jongman and Korsten 2016; Richter et al. 2021). This indicates irrigation water may serve as a source of contamination and suggests a possible route of transmission pathway of opportunistic pathogenic bacteria. *K. pneumoniae* isolates from irrigation water and vegetables on different farms showed 72% clonal similarity suggesting that the presence of common contamination sources and certain bacterial species are widely disseminated under diverse conditions. While in the present study, 76% similarity was observed in *K. pneumoniae* isolates from market. This indicates the presence of cross contamination during transportation, packaging, storage, and distribution chain. *K. aerogenes* isolated from vegetables on the same farms exhibited more related DNA fingerprinting patterns at 70% similarity. The results from the current findings are comparable to the clonal relatedness reported in for NDM‐5‐ producing *K. aerogenes* isolates (80%) in previous studies (Pan et al. 2021). Overall, clonal relatedness observed among isolates from vegetables, soil and irrigation water suggests potential common source of contamination across vegetable farming practices. ERIC‐PCR DNA fingerprinting patterns showed no specific association with antimicrobial resistance profile among isolates, which is in line with the findings of Richter et al. (2021).

As a limitation, this study only focused on screening limited resistance genetic markers not the entire resistome. Besides, ERIC‐PCR methods have limited discriminatory power. Therefore, future investigations should adopt high resolution techniques like WGS to generate accurate information regarding relatedness of closely alike isolates throughout the vegetable supply chain and public health interventions.

## 5 Conclusion

Irrigation water and soil are the pivotal reservoirs for gram‐negative pathogens. They can serve as a vehicle for dissemination of those bacteria to vegetables. The current findings provided significant baseline data about the occurrence of antimicrobial resistance and resistance genes among the Enterobacteriaceae family and *Acinetobacter* spp. in the agricultural environment and vegetables at the farm and market. The findings underscore the contribution of river irrigation water as a potential source and pathway for transmission of opportunistic human pathogenic bacteria to vegetables and humans through food chains. Therefore, appropriate treatment of river water before irrigation is recommended to reduce the spread of antimicrobial resistance to opportunistic pathogenic microorganisms.

## Author Contributions

**Hemen Tesfaye:** conceptualization, investigation, writing – original draft, methodology, validation, visualization, writing – review and editing, software, formal analysis, data curation, resources. **Adey Feleke Desta:** conceptualization, investigation, writing – original draft, methodology, writing – review and editing, resources, supervision. **Ahu Reis:** investigation. **Mine Egin:** investigation. **Osman Birol Ozgumus:** investigation, writing – review and editing, supervision. **Neslihan Akarsu:** investigation. **Mujib Abdulkadir:** supervision, investigation. **Haile Alemayehu:** investigation, resources, supervision. **Tadesse Eguale:** investigation, methodology, formal analysis, data curation, supervision, resources, writing – review and editing, writing – original draft. **Ali Osman Kilic:** conceptualization, investigation, writing – original draft, methodology, writing – review and editing, resources, supervision.

## Funding

The authors declare that no external funding was received for this research and publication of this article. Addis Ababa University provided partial institutional support.

## Conflicts of Interest

The authors declare no conflicts of interest.

## Supporting information


**Table S1:** The primer sequences used for the detection of antimicrobial resistance genes in Enterobacteriaceae family and *Acinetobacter* spp. isolates.
**Table S2:** *E. coli* ID corresponding to numbers in dendrogram Figure 4.
**Table S3:** *K. pneumoniae* ID corresponding to numbers in dendrogram Figure S1.
**Table S4:** *K. variicola*, *K. oxytoca*, and *E. asburiae* ID corresponding to numbers in dendrogram Figure S2.
**Table S5:** *A. baumannii, A. pittii, K. aerogenes*, and *E. bugandensis* ID corresponding to numbers in dendrogram Figure S3.
**Table S6:** *E. cloacae*, *E. kobei*, and *C. braakii* ID corresponding to numbers in dendrogram Figure S4.
**Figure S1:** Dendrogram showing fingerprinting profiles of *K. pneumoniae* phenotypically ESBL/AmpC and carbapenems producer strains isolated from Farms and supermarkets (vegetable, soil, and irrigation water samples). The profiles were obtained with ERIC‐PCR.
**Figure S2:** The dendrogram fingerprinting profiles of *E. asburiae* , *K. variicola*, and *K. oxytoca* phenotypically ESBL/AmpC and carbapenems producer strains isolated from Farms and supermarkets (vegetable, soil, and irrigation water samples). The profiles were obtained with ERIC‐PCR.
**Figure S3:** Dendrogram fingerprinting profiles of *A. baumannii* , *A. pittii* , *K. aerogenes* , and *E. bugandensis* phenotypically ESBL/AmpC and carbapenems producer strains isolated from Farms and markets (vegetable, soil, and irrigation water samples). The profiles were obtained with ERIC‐PCR.
**Figure S4:** Dendrogram fingerprinting profiles of *E. cloacae* , *E. kobei*, and *C. braakii* phenotypically ESBL/AmpC and carbapenems producer strains isolated from Farms and supermarkets (vegetable and soil samples). The profiles were obtained with ERIC‐PCR.
**Figure S5:** (A) Amplification of the donor and transconjugant ESBL and Carbapenemases encoded genes. M: “1 kb Thermo DNA ladder,” 1: Donor ETKP 42, 2: TXG ETKP 42, 3: Donor ETECO 160, 4: TXG ETECO 160, 5: Donor ETECO 169, 6: TXG ETECO 169, 7: Donor ETECO 169, 8: TXG ETECO 169, 9: Donor ETECO 200 10: TXG ETECO 200 11: Donor 42 12: TXG 42. (B) Plasmid DNA profiles of donor and transconjugant strains of *E. coli* J53‐2, alongside *E. coli* V517 as a reference plasmid strain. Note: M = Plasmid marker ( *E. coli* V517), 1: TXG *K. pneumoniae* (ETKP 42), 2: donor *K. pneumoniae* (ETKP 42), 3, TXG *C. braakii* (ETCB 160), 4: donor *C. braakii* (ETCB 160), 5: TXG *E. coli* (ETECO 169), 6: donor *E. coli* (ETECO 169), 7: TXG *E. coli* (ETECO 200), 8: donor *E. coli* (ETECO 200).

## Data Availability

The authors confirm that all the data are included in the manuscript.
