# Supplementary material for: Antimicrobial‐Resistance Genetic Markers Among Multidrug‐Resistant Enterobacteriaceae and Acinetobacter spp. From Vegetable Market Chains in Ethiopia
Source: Food Sci Nutr. 2026 Apr 12;14(4):e71761. doi: 10.1002/fsn3.71761 (PMC13071083; doi:10.1002/fsn3.71761)
Supplement: Supplementary file 1 — Table S1: The primer sequences used for the detection of antimicrobial resistance genes in Enterobacteriaceae family and Acinetobacter spp. isolates. Table S2: E. coli ID corresponding to numbers in dendrogram Figure 4. Table S3: K. pneumoniae ID corresponding to numbers in dendrogram Figure S1. Table S4: K. variicola, K. oxytoca, and E. asburiae ID corresponding to numbers in dendrogram Figure S2. Table S5: A. baumannii, A. pittii, K. aerogenes , and E. bugandensis ID corresponding to numbers in dendrogram Figure S3. Table S6: E. cloacae, E. kobei, and C. braakii ID corresponding to numbers in dendrogram Figure S4. Figure S1: Dendrogram showing fingerprinting profiles of K. pneumoniae phenotypically ESBL/AmpC and carbapenems producer strains isolated from Farms and supermarkets (vegetable, soil, and irrigation water samples). The profiles were obtained with ERIC‐PCR. Figure S2: The dendrogram fingerprinting profiles of E. asburiae , K. variicola, and K. oxytoca phenotypically ESBL/AmpC and carbapenems producer strains isolated from Farms and supermarkets (vegetable, soil, and irrigation water samples). The profiles were obtained with ERIC‐PCR. Figure S3: Dendrogram fingerprinting profiles of A. baumannii , A. pittii , K. aerogenes , and E. bugandensis phenotypically ESBL/AmpC and carbapenems producer strains isolated from Farms and markets (vegetable, soil, and irrigation water samples). The profiles were obtained with ERIC‐PCR. Figure S4: Dendrogram fingerprinting profiles of E. cloacae , E. kobei, and C. braakii phenotypically ESBL/AmpC and carbapenems producer strains isolated from Farms and supermarkets (vegetable and soil samples). The profiles were obtained with ERIC‐PCR. Figure S5: (A) Amplification of the donor and transconjugant ESBL and Carbapenemases encoded genes. M: “1 kb Thermo DNA ladder,” 1: Donor ETKP 42, 2: TXG ETKP 42, 3: Donor ETECO 160, 4: TXG ETECO 160, 5: Donor ETECO 169, 6: TXG ETECO 169, 7: Donor ETECO 169, 8: TXG ETECO 169, 9: Donor ETECO [file FSN3-14-e71761-s001.zip › figure captions.docx]

**Supplementary materials:** the supplementary materials include Table S1-S6 and Figure S1-S5. **Table S1** provides detailed the primer sequences used for the detection of antimicrobial resistance genes in Enterobacteriaceae family and *Acinetobacter* spp. isolates. **Table S2:** describes *E. coli* ID corresponding to numbers in dendrogram Figure 4. **Table S3:** tells *K. pneumoniae* ID corresponding to numbers in dendrogram Figure S1. **Table S4:** explain *K. variicola, K. oxytoca and E. asburiae* ID corresponding to numbers in dendrogram Figure S2. **Table S5:** describes *A. baumannii, A. pittii, K. aerogenes*, and *E. bugandensis* ID corresponding to numbers in dendrogram Figure S3. **Table S6:** explains *E. cloacae, E. kobei and C. braakii* ID corresponding to numbers in dendrogram Figure S4.

**Figure S1** Dendrogram showing fingerprinting profiles of *K. pneumoniae* phenotypically ESBL/AmpC and carbapenems producer strains isolated from Farms and markets (vegetable, soil and irrigation water samples). The profiles were obtained with ERIC-PCR. **Figure S2** describes the dendrogram fingerprinting profiles of *E. asburiae, K. variicola and K. oxytoca* phenotypically ESBL/AmpC and carbapenems producer strains isolated from Farms and markets (vegetable, soil and irrigation water samples). The profiles were obtained with ERIC-PCR. **Figure S3** shows dendrogram fingerprinting profiles of *A. baumannii*, *A. pittii*, *K. aerogenes*, and *E. bugandensis* phenotypically ESBL/AmpC and carbapenems producer strains isolated from Farms and markets (vegetable, soil and irrigation water samples). The profiles were obtained with ERIC-PCR, while **Figure S4** presents dendrogram fingerprinting profiles of *E. cloacae, E. kobei* and *C. braakii* phenotypically ESBL/AmpC and carbapenems producer strains isolated from Farms and markets (vegetable, and soil samples). The profiles were obtained with ERIC-PCR. Furthermore, **Figure S5** presents (A) Amplification of the donor and transconjugant ESBL and Carbapenemases encoded genes. M: “1kb Thermo DNA ladder “, 1: Donor ETKP 42, 2: TXG ETKP 42, 3: Donor ETECO 160, 4: TXG ETECO 160, 5: Donor ETECO 169, 6: TXG ETECO 169, 7: Donor ETECO 169, 8: TXG ETECO 169, 9: Donor ETECO 200 10: TXG ETECO 200 11: Donor 42 12: TXG 42. (B) Plasmid DNA profiles of donor and transconjugant strains of *E. coli* J53-2, alongside *E. coli* V517 as a reference plasmid strain. Note: M= Plasmid marker (*E. coli* V517), 1: TXG *K. pneumoniae* (ETKP 42), 2: donor *K. pneumoniae* (ETKP 42), 3, TXG *C. braakii* (ETCB 160), 4: donor *C. braakii* (ETCB 160), 5: TXG *E. coli* (ETECO 169), 6: donor *E. coli* (ETECO 169), 7: TXG *E. coli* (ETECO 200), 8: donor *E. coli* (ETECO 200).
