# Supplementary material for: Antimicrobial‐Resistance Genetic Markers Among Multidrug‐Resistant Enterobacteriaceae and Acinetobacter spp. From Vegetable Market Chains in Ethiopia
Source: Food Sci Nutr. 2026 Apr 12;14(4):e71761. doi: 10.1002/fsn3.71761 (PMC13071083; doi:10.1002/fsn3.71761)
Supplement: Supplementary file 1 — Table S1: The primer sequences used for the detection of antimicrobial resistance genes in Enterobacteriaceae family and Acinetobacter spp. isolates. Table S2: E. coli ID corresponding to numbers in dendrogram Figure 4. Table S3: K. pneumoniae ID corresponding to numbers in dendrogram Figure S1. Table S4: K. variicola, K. oxytoca, and E. asburiae ID corresponding to numbers in dendrogram Figure S2. Table S5: A. baumannii, A. pittii, K. aerogenes , and E. bugandensis ID corresponding to numbers in dendrogram Figure S3. Table S6: E. cloacae, E. kobei, and C. braakii ID corresponding to numbers in dendrogram Figure S4. Figure S1: Dendrogram showing fingerprinting profiles of K. pneumoniae phenotypically ESBL/AmpC and carbapenems producer strains isolated from Farms and supermarkets (vegetable, soil, and irrigation water samples). The profiles were obtained with ERIC‐PCR. Figure S2: The dendrogram fingerprinting profiles of E. asburiae , K. variicola, and K. oxytoca phenotypically ESBL/AmpC and carbapenems producer strains isolated from Farms and supermarkets (vegetable, soil, and irrigation water samples). The profiles were obtained with ERIC‐PCR. Figure S3: Dendrogram fingerprinting profiles of A. baumannii , A. pittii , K. aerogenes , and E. bugandensis phenotypically ESBL/AmpC and carbapenems producer strains isolated from Farms and markets (vegetable, soil, and irrigation water samples). The profiles were obtained with ERIC‐PCR. Figure S4: Dendrogram fingerprinting profiles of E. cloacae , E. kobei, and C. braakii phenotypically ESBL/AmpC and carbapenems producer strains isolated from Farms and supermarkets (vegetable and soil samples). The profiles were obtained with ERIC‐PCR. Figure S5: (A) Amplification of the donor and transconjugant ESBL and Carbapenemases encoded genes. M: “1 kb Thermo DNA ladder,” 1: Donor ETKP 42, 2: TXG ETKP 42, 3: Donor ETECO 160, 4: TXG ETECO 160, 5: Donor ETECO 169, 6: TXG ETECO 169, 7: Donor ETECO 169, 8: TXG ETECO 169, 9: Donor ETECO [file FSN3-14-e71761-s001.zip › Supplementary Table S1-S6.docx]

Table S1: The Primer sequences used for the detection of antimicrobial resistance genes in Enterobacteriaceae family and Acinetobacter spp. isolates.

| **Antimicrobial** | **Target**  **Genes** | **Primer Sequences (5’🡪3’)** | **Annealing**  **temp (°C)** | **Length**  **(bp)** | **Thermocycling conditions** | **Reference** |
| --- | --- | --- | --- | --- | --- | --- |
| Carbapenem | *bla_NDM_* | F: GAGATTGCCGAGCGACTTG  R: CGAATGTCTGGCAGCACACTT | 57 | 497 | 94^o^C for 3 min, 35 cycle, 94^o^C for 30s, 57 for 30s, 72^o^C for 1min, 72^o^C for 10min. | (Cicek *et al*., 2014) |
|  | *bla_VIM_* | F : GATGGTGTTTGGTCGCATA  R : CGAATGCGCAGCACCAG | 52 | 390 | 94^o^C for 10 min, 36 cycles, 94^o^C for 30s, 52^o^C for 40s, 72^o^C for 50s, 72^o^C for 5min | (Poirel *et al*., 2011) |
|  | *bla_KPC_* | F: ATGTCACTGTATCGCCGTCT  R: TTTTCAGAGCCTTACTGCCC | 55 | 893 | 94^o^C for 5min, 30 cycle, 94^o^C for 30s, 55^o^C for 45s, 72^o^C for 1min, 72^o^C for 10min | (Schechner *et al*., 2009) |
|  | *bla_IMP_* | F: GGAATAGAGTGGCTTAAYTCTC  R: GGTTTAAYAAAACAACCACC | 56 | 232 | 95^o^C for 3min, 30 cycles, 95^o^C for 30s, 56^o^C for 30s, 72^o^C for 45s, 72^o^C for 5min | (Hatrongjit *et al.,* 2018) |
|  | *bla_OXA-48_* | F: TTGGTGGCATCGATTATCGG  R: GAGCACTTCTTTTGTGATGGC | 60 | 743 | 95^o^C for 5min, 32 cycles of 95^o^C for 30s, 60^o^C for 30s, and 72^o^C for 30s and 72^o^C for 10 min | (Poirel, Bonnin, & Nordmann, 2012) |
| *β*-lactamases | *bla_TEM_* | F: ATGAGTATTCAACATTTCCG  R: CTGACAGTTACCAATGCTTA | 55 | 867 | 94^o^C for 5min, 30 cycle, 94^o^C 1min, 55^o^C for 1 min, 72^o^C for 1 min, 72^o^C 10min | (Lim *et al.,* 2009) |
|  | *bla_SHV_* | F: TCA GCG AAA AAC ACC TTG  R: TCC CGC AGA TAA ATC ACC A | 55 | 475 | 94^o^C for 5min, 30 cycles, 94^o^C for 30s, 55^o^C for 30s, 72^o^C for 60s, 72^o^C for 10min | (Babini & Livermore, 2000) |
|  | *bla_CTX-M_* | F: CGCTTTGCGATGTGCAG  R: ACCGCGATATCGTTGGT | 55 | 550 | 94^o^C for 5min, 30cycle, 94^o^C for 1min, 55^o^C for 1min, 72^o^C for 1min, 72^o^C for 10min | (Poirel *et al.,* 2011) |
| Sulfonamides | *Sul1* | F: GGCCGATGAGATCAGACGTA  R: TTTGAAGGTTCGACAGCACG | 58 | 413 | 94^o^C for 3min, 30 cycle, 94^o^C for 1min, 58^o^C for 1min, 72^o^C for 1min, 72^o^C for 10min | (Jiang *et al.,* 2019) |
|  | *Sul2* | F: GCGCTCAAGGCAGATGGCATT  R: GCGTTTGATACCGGCACCCGT | 58 | 793 | 94^o^C for 3min, 30 cycles, 94^o^C for 30s, 58^o^C for 45s, 72^o^C for 1min, 72^o^C for 7min | (Qiu *et al.,* 2019) |
| quinolone | *qnrA* | F: ATTTCTCACGCCAGGATTTG  R: GATCGGCAAAGGTTAGGTCA | 58 | 516 | 94^o^C for 2min, 34 cycle, 94^o^C for 45s, 58^o^C for 1min, 72^o^C for 1min, 72^o^C for 5min | (Robicsek *et al*., 2006) |
|  | *qnrB* | F: GATCGTGAAAGCCAGAAAGG  R: ACGATGCCTGGTAGTTGTCC | 58 | 469 | 94^o^C for 2min, 34 cycle, 94^o^C for 45s, 58^o^C for 1min, 72^o^C for 1min, 72^o^C for 5min | (Robicsek *et al*., 2006) |
|  | *qnrS* | F: GCAAGTTCATTGAACAGGGT  R: TCTAAACCGTCGAGTTCGGCG | 58 | 428 | 94^o^C for 2min, 34 cycle, 94^o^C for 45s, 58^o^C for 1min, 72^o^C for 1min, 72^o^C for 5min | (El-Badawy *et al*., 2017) |
| Tetracycline | *tetA* | F: GTAATTCTGAGCACTGTCGC  R: CTGCCTGGACAACATTGCTT | 58 | 917 | 94^o^C for 3min, 30 cycle, 94^o^C for 45s, 58^o^C for 45s, 72^o^C for 1min, 72^o^C for 7min | (Guardabassi *et al*., 2000) |
|  | *tetB* | F: CTCAGTATTCCAAGCCTTTG  R: ACTCCCCTGAGCTTGAGGGG | 58 | 396 | 94^o^C for 3min, 30 cycle, 94^o^C for 45s, 58 ^o^C for 45s, 72^o^C for 1min, 72^o^C for 7min | (Guardabassi *et al.*, 2000) |
| Cefotaxime and Ampicilin | *AmpC* | F: CCCCGCTTATAGAGCAACAA  R: TCAATGGTCGACTTCACACC | 60 | 634 | 94^o^C for 5min, 2cycles at 94^o^C for 7min, 60^o^C for 5min, 72^o^C for 1min, 30 cycles 94^o^C for 1min, 60^o^C for 2min, 72^o^C for 1min, 72^o^C for 10min | (Paul-Satyaseela *et al.*, 2016) |

Table S2: *E. coli* ID corresponding to numbers in dendrogram Figure 4.

| **No** **top-to-bottom order** | **Strain ID** |
| --- | --- |
| 8 | ETECO175 |
| 14 | ETECO201 |
| 13 | ETECO200 |
| 21 | ETECO226 |
| 9 | ETECO169 |
| 7 | ETECO182 |
| 11 | ETECO180 |
| 15 | ETECO214 |
| 5 | ETECO225 |
| 6 | ETECO203 |
| 4 | ETECO212 |
| 12 | ETECO219 |
| 18 | ETECO207 |
| 3 | ETECO211 |
| 20 | ETECO 177 |
| 19 | ETECO221 |
| 17 | ETECO184 |
| 22 | ETECO185 |
| 16 | ETECO183 |
| 10 | ETECO168 |
| 2 | ETECO187 |

Table S3: *K. pneumoniae* ID corresponding to numbers in dendrogram Figure S1.

| **No top-to-bottom order** | **Strain ID** |
| --- | --- |
| 1 | ETKP24 |
| 2 | ETKP26 |
| 3 | ETKP31 |
| 4 | ETKP32 |
| 5 | ETKP33 |
| 6 | ETKP36 |
| 7 | ETKP35 |
| 8 | ETKP37 |
| 9 | ETKP41 |
| 10 | ETKP42 |
| 11 | ETKP43 |
| 12 | ETKP56 |
| 13 | ETKP57 |
| 14 | ETKP61 |
| 15 | ETKP58 |

Table S4: *K. variicola*, *K. oxytoca* and *E. asburiae* ID corresponding to numbers in dendrogram Figure S2.

| **No top-to-bottom order** | **Strain ID** |
| --- | --- |
| 9 | ETKV71 |
| 4 | ETKV73 |
| 3 | ETKV75 |
| 10 | ETKO87 |
| 7 | ETEA103 |
| 6 | ETEA108 |
| 8 | ETEA117 |
| 5 | ETEA111 |
| 11 | ETEA121 |
| 2 | ETEA270 |

Table S5: *A. baumannii, A. pittii, K. aerogenes, and E. bugandensis* ID corresponding to numbers in dendrogram Figure S3.

| **No top-to-bottom order** | **Strain ID** |
| --- | --- |
| 4 | ETAP6 |
| 14 | ETAP8 |
| 11 | ETAP17 |
| 9 | ETAP18 |
| 6 | ETAB2 |
| 12 | ETAB3 |
| 13 | ETAB5 |
| 10 | ETEB99 |
| 8 | ETEB101 |
| 7 | ETEB137 |
| 5 | ETEA65 |
| 3 | ETKA66 |
| 2 | ETKA67 |

Table S6: *E. cloacae, E. kobei and C. braakii* ID corresponding to numbers in dendrogram Figure S4.

| **No top-to-bottom order** | **Strain ID** |
| --- | --- |
| 8 | ETEC97 |
| 6 | ETEC98 |
| 5 | ETEC131 |
| 7 | ETEK126 |
| 4 | ETEK127 |
| 3 | ETEK129 |
| 2 | ETCB160 |

**References**

Babini, G., & Livermore, D. (2000). Are SHV beta-lactamases universal in Klebsiella pneumoniae? . *Antimicrob Agents Chemother. 44(8):2230* <https://doi.org/doi:10.1128/AAC.44.8.2230-2230.2000>.

Cicek, A. C., Saral, A., Iraz, M., Ceylan, A., Duzgun, A. O., Peleg, A. Y., & Sandalli, C. (2014). OXA- and GES-type beta-lactamases predominate in extensively drug-resistant Acinetobacter baumannii isolates from a Turkish University Hospital. *Clin Microbiol Infect*, *20*(5), 410-415. <https://doi.org/10.1111/1469-0691.12338>

El-Badawy, M. F., Tawakol, W. M., El-Far, S. W., Maghrabi, I. A., Al-Ghamdi, S. A., Mansy, M. S., Ashour, M. S., & Shohayeb, M. M. (2017). Molecular Identification of Aminoglycoside-Modifying Enzymes and Plasmid-Mediated Quinolone Resistance Genes among Klebsiella pneumoniae Clinical Isolates Recovered from Egyptian Patients. *Int J Microbiol*, *2017*, 8050432. <https://doi.org/10.1155/2017/8050432>

Guardabassi, L., Dijkshoorn, L., Collard, J. M., Olsen, J. E., & Dalsgaard, A. (2000). Distribution and in-vitro transfer of tetracycline resistance determinants in clinical and aquatic Acinetobacter strains. *J Med Microbiol*, *49*(10), 929-936. <https://doi.org/10.1099/0022-1317-49-10-929>

Hatrongjit, R., Kerdsin, A., Akeda, Y., & Hamada, S. (2018). Detection of plasmid-mediated colistin-resistant and carbapenem-resistant genes by multiplex PCR. *MethodsX*, *5*, 532-536. <https://doi.org/10.1016/j.mex.2018.05.016>

Jiang, H., Cheng, H., Liang, Y., Yu, S., Yu, T., Fang, J., & Zhu, C. (2019). Diverse Mobile Genetic Elements and Conjugal Transferability of Sulfonamide Resistance Genes (sul1, sul2, and sul3) in Escherichia coli Isolates From Penaeus vannamei and Pork From Large Markets in Zhejiang, China. *Front Microbiol*, *10*, 1787. <https://doi.org/10.3389/fmicb.2019.01787>

Lim, K. T., Yasin, R., Yeo, C. C., Puthucheary, S., & Thong, K. L. (2009). Characterization of multidrug resistant ESBL-producing Escherichia coli isolates from hospitals in Malaysia. *J Biomed Biotechnol*, *2009*, 165637. <https://doi.org/10.1155/2009/165637>

Paul-Satyaseela, M., Murali, S., Thirunavukkarasu, B., Naraharirao, M. H., & Jambulingam, M. (2016). Characterization of Antibiotic Resistance Profiles of Ocular Enterobacteriaceae Isolates. *Eur J Microbiol Immunol (Bp)*, *6*(1), 40-48. <https://doi.org/10.1556/1886.2015.00047>

Poirel, L., Bonnin, R. A., & Nordmann, P. (2012). Genetic features of the widespread plasmid coding for the carbapenemase OXA-48. *Antimicrob Agents Chemother*, *56*(1), 559-562. <https://doi.org/10.1128/AAC.05289-11>

Poirel, L., Walsh, T. R., Cuvillier, V., & Nordmann, P. (2011). Multiplex PCR for detection of acquired carbapenemase genes. *Diagn Microbiol Infect Dis*, *70*(1), 119-123. <https://doi.org/10.1016/j.diagmicrobio.2010.12.002>

Qiu, J., Jiang, Z., Ju, Z., Zhao, X., Yang, J., Guo, H., & Sun, S. (2019). Molecular and Phenotypic Characteristics ofEscherichia coliIsolates from Farmed Minks in Zhucheng, China. *BioMed Research International*, *2019*, 1-12. <https://doi.org/10.1155/2019/3917841>

Robicsek, A., Strahilevitz, J., Sahm, D. F., Jacoby, G. A., & Hooper, D. C. (2006). qnr prevalence in ceftazidime-resistant Enterobacteriaceae isolates from the United States. *Antimicrob Agents Chemother*, *50*(8), 2872-2874. <https://doi.org/10.1128/AAC.01647-05>

Schechner, V., Straus-Robinson, K., Schwartz, D., Pfeffer, I., Tarabeia, J., Moskovich, R., Chmelnitsky, I., Schwaber, M. J., Carmeli, Y., & Navon-Venezia, S. (2009). Evaluation of PCR-Based Testing for Surveillance of KPC-Producing Carbapenem-Resistant Members of the Enterobacteriaceae Family. *Journal of Clinical Microbiology*, *47*(10), 3261-3265. <https://doi.org/10.1128/jcm.02368-08>
