# Supplementary figures and images for: Antimicrobial‐Resistance Genetic Markers Among Multidrug‐Resistant Enterobacteriaceae and Acinetobacter spp. From Vegetable Market Chains in Ethiopia
Source: Food Sci Nutr. 2026 Apr 12;14(4):e71761. doi: 10.1002/fsn3.71761 (PMC13071083; doi:10.1002/fsn3.71761)

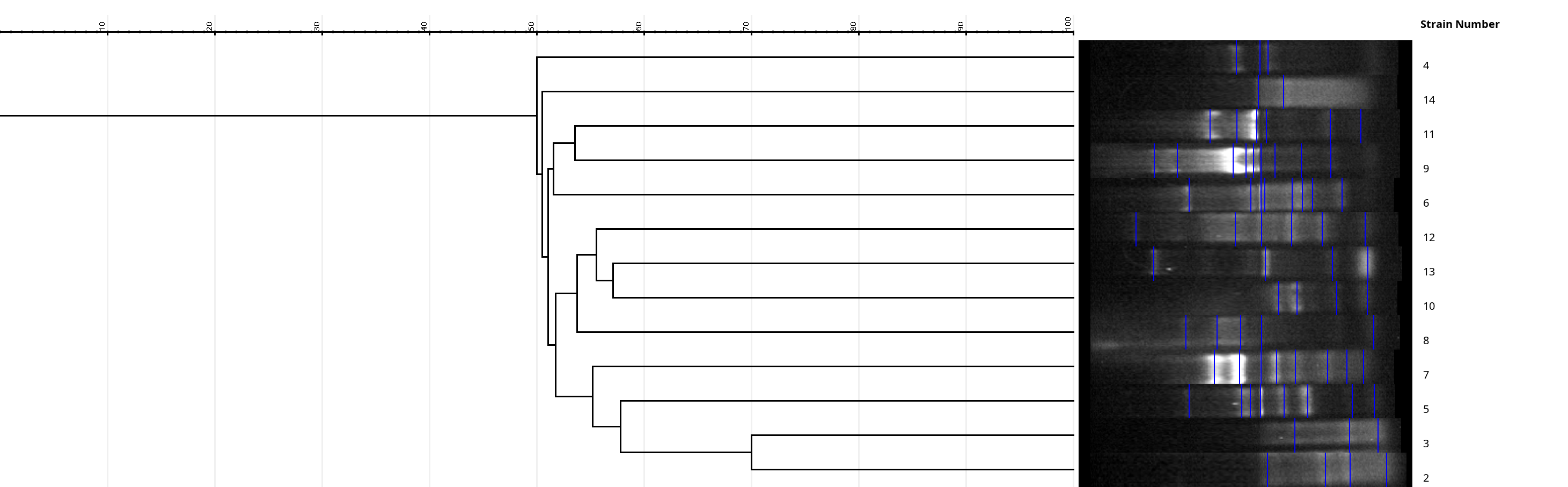

Supplement: Supplementary file 1 — Table S1: The primer sequences used for the detection of antimicrobial resistance genes in Enterobacteriaceae family and Acinetobacter spp. isolates. Table S2: E. coli ID corresponding to numbers in dendrogram Figure 4. Table S3: K. pneumoniae ID corresponding to numbers in dendrogram Figure S1. Table S4: K. variicola, K. oxytoca, and E. asburiae ID corresponding to numbers in dendrogram Figure S2. Table S5: A. baumannii, A. pittii, K. aerogenes , and E. bugandensis ID corresponding to numbers in dendrogram Figure S3. Table S6: E. cloacae, E. kobei, and C. braakii ID corresponding to numbers in dendrogram Figure S4. Figure S1: Dendrogram showing fingerprinting profiles of K. pneumoniae phenotypically ESBL/AmpC and carbapenems producer strains isolated from Farms and supermarkets (vegetable, soil, and irrigation water samples). The profiles were obtained with ERIC‐PCR. Figure S2: The dendrogram fingerprinting profiles of E. asburiae , K. variicola, and K. oxytoca phenotypically ESBL/AmpC and carbapenems producer strains isolated from Farms and supermarkets (vegetable, soil, and irrigation water samples). The profiles were obtained with ERIC‐PCR. Figure S3: Dendrogram fingerprinting profiles of A. baumannii , A. pittii , K. aerogenes , and E. bugandensis phenotypically ESBL/AmpC and carbapenems producer strains isolated from Farms and markets (vegetable, soil, and irrigation water samples). The profiles were obtained with ERIC‐PCR. Figure S4: Dendrogram fingerprinting profiles of E. cloacae , E. kobei, and C. braakii phenotypically ESBL/AmpC and carbapenems producer strains isolated from Farms and supermarkets (vegetable and soil samples). The profiles were obtained with ERIC‐PCR. Figure S5: (A) Amplification of the donor and transconjugant ESBL and Carbapenemases encoded genes. M: “1 kb Thermo DNA ladder,” 1: Donor ETKP 42, 2: TXG ETKP 42, 3: Donor ETECO 160, 4: TXG ETECO 160, 5: Donor ETECO 169, 6: TXG ETECO 169, 7: Donor ETECO 169, 8: TXG ETECO 169, 9: Donor ETECO [file FSN3-14-e71761-s001.zip › dendrogram_ FS3 A. baumannii, A. pittii.tif]

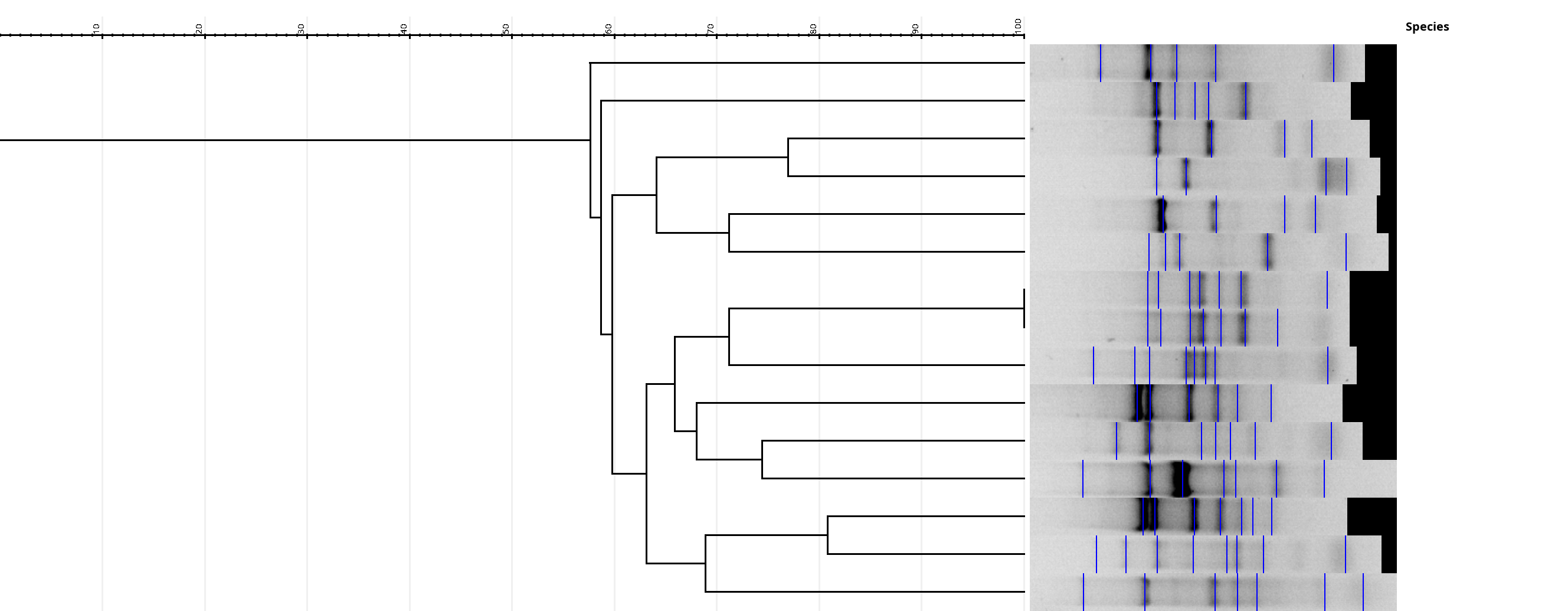

Supplement: Supplementary file 1 — Table S1: The primer sequences used for the detection of antimicrobial resistance genes in Enterobacteriaceae family and Acinetobacter spp. isolates. Table S2: E. coli ID corresponding to numbers in dendrogram Figure 4. Table S3: K. pneumoniae ID corresponding to numbers in dendrogram Figure S1. Table S4: K. variicola, K. oxytoca, and E. asburiae ID corresponding to numbers in dendrogram Figure S2. Table S5: A. baumannii, A. pittii, K. aerogenes , and E. bugandensis ID corresponding to numbers in dendrogram Figure S3. Table S6: E. cloacae, E. kobei, and C. braakii ID corresponding to numbers in dendrogram Figure S4. Figure S1: Dendrogram showing fingerprinting profiles of K. pneumoniae phenotypically ESBL/AmpC and carbapenems producer strains isolated from Farms and supermarkets (vegetable, soil, and irrigation water samples). The profiles were obtained with ERIC‐PCR. Figure S2: The dendrogram fingerprinting profiles of E. asburiae , K. variicola, and K. oxytoca phenotypically ESBL/AmpC and carbapenems producer strains isolated from Farms and supermarkets (vegetable, soil, and irrigation water samples). The profiles were obtained with ERIC‐PCR. Figure S3: Dendrogram fingerprinting profiles of A. baumannii , A. pittii , K. aerogenes , and E. bugandensis phenotypically ESBL/AmpC and carbapenems producer strains isolated from Farms and markets (vegetable, soil, and irrigation water samples). The profiles were obtained with ERIC‐PCR. Figure S4: Dendrogram fingerprinting profiles of E. cloacae , E. kobei, and C. braakii phenotypically ESBL/AmpC and carbapenems producer strains isolated from Farms and supermarkets (vegetable and soil samples). The profiles were obtained with ERIC‐PCR. Figure S5: (A) Amplification of the donor and transconjugant ESBL and Carbapenemases encoded genes. M: “1 kb Thermo DNA ladder,” 1: Donor ETKP 42, 2: TXG ETKP 42, 3: Donor ETECO 160, 4: TXG ETECO 160, 5: Donor ETECO 169, 6: TXG ETECO 169, 7: Donor ETECO 169, 8: TXG ETECO 169, 9: Donor ETECO [file FSN3-14-e71761-s001.zip › dendrogram_FS1_Klebsiella pneumoniae.tif]

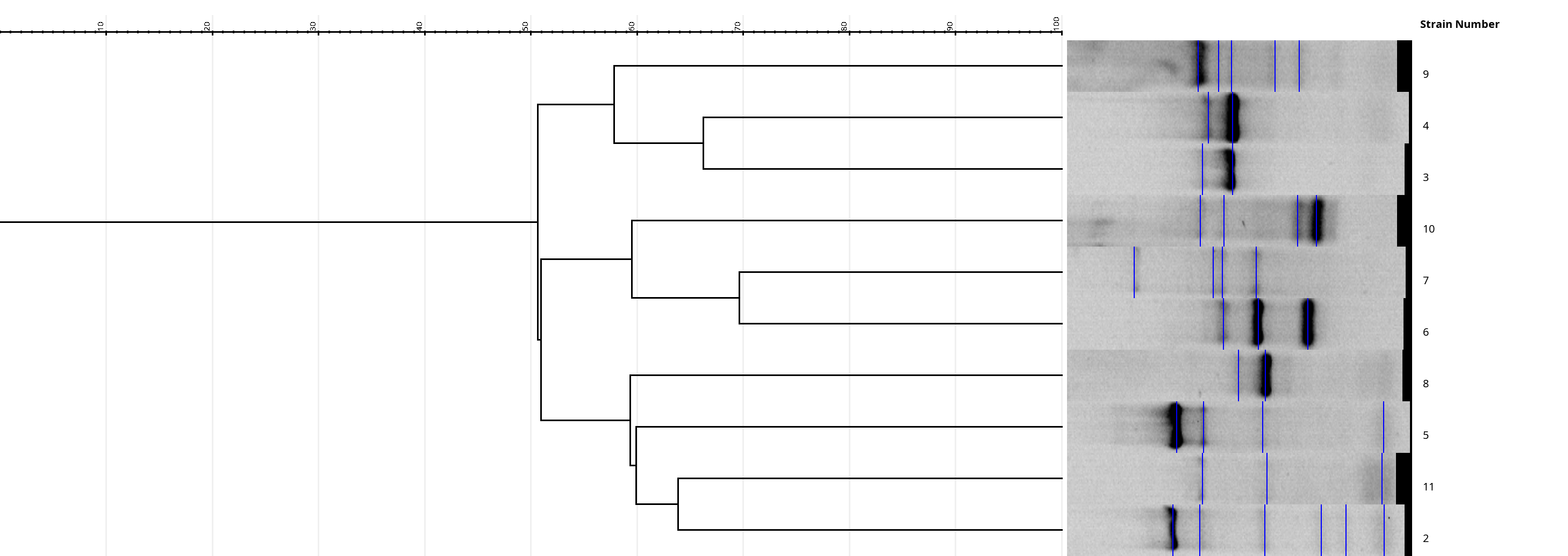

Supplement: Supplementary file 1 — Table S1: The primer sequences used for the detection of antimicrobial resistance genes in Enterobacteriaceae family and Acinetobacter spp. isolates. Table S2: E. coli ID corresponding to numbers in dendrogram Figure 4. Table S3: K. pneumoniae ID corresponding to numbers in dendrogram Figure S1. Table S4: K. variicola, K. oxytoca, and E. asburiae ID corresponding to numbers in dendrogram Figure S2. Table S5: A. baumannii, A. pittii, K. aerogenes , and E. bugandensis ID corresponding to numbers in dendrogram Figure S3. Table S6: E. cloacae, E. kobei, and C. braakii ID corresponding to numbers in dendrogram Figure S4. Figure S1: Dendrogram showing fingerprinting profiles of K. pneumoniae phenotypically ESBL/AmpC and carbapenems producer strains isolated from Farms and supermarkets (vegetable, soil, and irrigation water samples). The profiles were obtained with ERIC‐PCR. Figure S2: The dendrogram fingerprinting profiles of E. asburiae , K. variicola, and K. oxytoca phenotypically ESBL/AmpC and carbapenems producer strains isolated from Farms and supermarkets (vegetable, soil, and irrigation water samples). The profiles were obtained with ERIC‐PCR. Figure S3: Dendrogram fingerprinting profiles of A. baumannii , A. pittii , K. aerogenes , and E. bugandensis phenotypically ESBL/AmpC and carbapenems producer strains isolated from Farms and markets (vegetable, soil, and irrigation water samples). The profiles were obtained with ERIC‐PCR. Figure S4: Dendrogram fingerprinting profiles of E. cloacae , E. kobei, and C. braakii phenotypically ESBL/AmpC and carbapenems producer strains isolated from Farms and supermarkets (vegetable and soil samples). The profiles were obtained with ERIC‐PCR. Figure S5: (A) Amplification of the donor and transconjugant ESBL and Carbapenemases encoded genes. M: “1 kb Thermo DNA ladder,” 1: Donor ETKP 42, 2: TXG ETKP 42, 3: Donor ETECO 160, 4: TXG ETECO 160, 5: Donor ETECO 169, 6: TXG ETECO 169, 7: Donor ETECO 169, 8: TXG ETECO 169, 9: Donor ETECO [file FSN3-14-e71761-s001.zip › dendrogram_FS2_E. asburiae, K. variicola.tif]

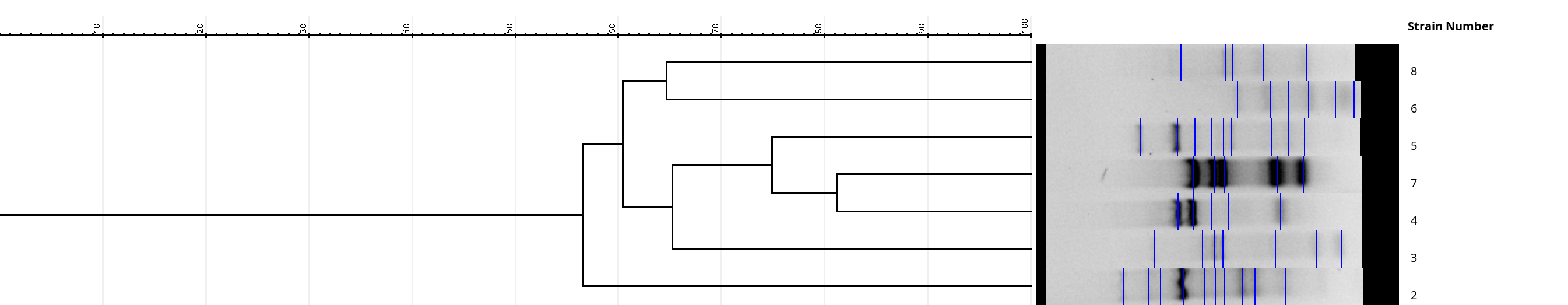

Supplement: Supplementary file 1 — Table S1: The primer sequences used for the detection of antimicrobial resistance genes in Enterobacteriaceae family and Acinetobacter spp. isolates. Table S2: E. coli ID corresponding to numbers in dendrogram Figure 4. Table S3: K. pneumoniae ID corresponding to numbers in dendrogram Figure S1. Table S4: K. variicola, K. oxytoca, and E. asburiae ID corresponding to numbers in dendrogram Figure S2. Table S5: A. baumannii, A. pittii, K. aerogenes , and E. bugandensis ID corresponding to numbers in dendrogram Figure S3. Table S6: E. cloacae, E. kobei, and C. braakii ID corresponding to numbers in dendrogram Figure S4. Figure S1: Dendrogram showing fingerprinting profiles of K. pneumoniae phenotypically ESBL/AmpC and carbapenems producer strains isolated from Farms and supermarkets (vegetable, soil, and irrigation water samples). The profiles were obtained with ERIC‐PCR. Figure S2: The dendrogram fingerprinting profiles of E. asburiae , K. variicola, and K. oxytoca phenotypically ESBL/AmpC and carbapenems producer strains isolated from Farms and supermarkets (vegetable, soil, and irrigation water samples). The profiles were obtained with ERIC‐PCR. Figure S3: Dendrogram fingerprinting profiles of A. baumannii , A. pittii , K. aerogenes , and E. bugandensis phenotypically ESBL/AmpC and carbapenems producer strains isolated from Farms and markets (vegetable, soil, and irrigation water samples). The profiles were obtained with ERIC‐PCR. Figure S4: Dendrogram fingerprinting profiles of E. cloacae , E. kobei, and C. braakii phenotypically ESBL/AmpC and carbapenems producer strains isolated from Farms and supermarkets (vegetable and soil samples). The profiles were obtained with ERIC‐PCR. Figure S5: (A) Amplification of the donor and transconjugant ESBL and Carbapenemases encoded genes. M: “1 kb Thermo DNA ladder,” 1: Donor ETKP 42, 2: TXG ETKP 42, 3: Donor ETECO 160, 4: TXG ETECO 160, 5: Donor ETECO 169, 6: TXG ETECO 169, 7: Donor ETECO 169, 8: TXG ETECO 169, 9: Donor ETECO [file FSN3-14-e71761-s001.zip › dendrogram_FS4 E. cloacae, E. kobei.tif]

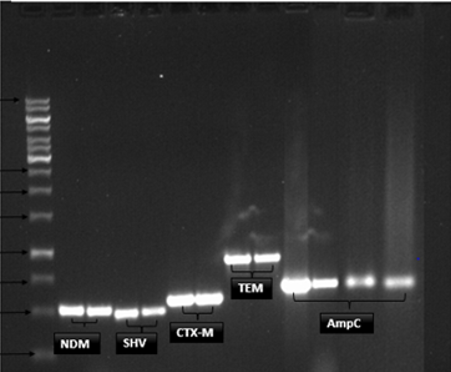

Supplement: Supplementary file 1 — Table S1: The primer sequences used for the detection of antimicrobial resistance genes in Enterobacteriaceae family and Acinetobacter spp. isolates. Table S2: E. coli ID corresponding to numbers in dendrogram Figure 4. Table S3: K. pneumoniae ID corresponding to numbers in dendrogram Figure S1. Table S4: K. variicola, K. oxytoca, and E. asburiae ID corresponding to numbers in dendrogram Figure S2. Table S5: A. baumannii, A. pittii, K. aerogenes , and E. bugandensis ID corresponding to numbers in dendrogram Figure S3. Table S6: E. cloacae, E. kobei, and C. braakii ID corresponding to numbers in dendrogram Figure S4. Figure S1: Dendrogram showing fingerprinting profiles of K. pneumoniae phenotypically ESBL/AmpC and carbapenems producer strains isolated from Farms and supermarkets (vegetable, soil, and irrigation water samples). The profiles were obtained with ERIC‐PCR. Figure S2: The dendrogram fingerprinting profiles of E. asburiae , K. variicola, and K. oxytoca phenotypically ESBL/AmpC and carbapenems producer strains isolated from Farms and supermarkets (vegetable, soil, and irrigation water samples). The profiles were obtained with ERIC‐PCR. Figure S3: Dendrogram fingerprinting profiles of A. baumannii , A. pittii , K. aerogenes , and E. bugandensis phenotypically ESBL/AmpC and carbapenems producer strains isolated from Farms and markets (vegetable, soil, and irrigation water samples). The profiles were obtained with ERIC‐PCR. Figure S4: Dendrogram fingerprinting profiles of E. cloacae , E. kobei, and C. braakii phenotypically ESBL/AmpC and carbapenems producer strains isolated from Farms and supermarkets (vegetable and soil samples). The profiles were obtained with ERIC‐PCR. Figure S5: (A) Amplification of the donor and transconjugant ESBL and Carbapenemases encoded genes. M: “1 kb Thermo DNA ladder,” 1: Donor ETKP 42, 2: TXG ETKP 42, 3: Donor ETECO 160, 4: TXG ETECO 160, 5: Donor ETECO 169, 6: TXG ETECO 169, 7: Donor ETECO 169, 8: TXG ETECO 169, 9: Donor ETECO [file FSN3-14-e71761-s001.zip › FS5 (A).png]

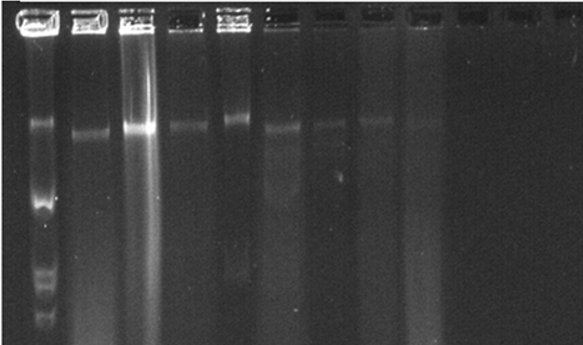

Supplement: Supplementary file 1 — Table S1: The primer sequences used for the detection of antimicrobial resistance genes in Enterobacteriaceae family and Acinetobacter spp. isolates. Table S2: E. coli ID corresponding to numbers in dendrogram Figure 4. Table S3: K. pneumoniae ID corresponding to numbers in dendrogram Figure S1. Table S4: K. variicola, K. oxytoca, and E. asburiae ID corresponding to numbers in dendrogram Figure S2. Table S5: A. baumannii, A. pittii, K. aerogenes , and E. bugandensis ID corresponding to numbers in dendrogram Figure S3. Table S6: E. cloacae, E. kobei, and C. braakii ID corresponding to numbers in dendrogram Figure S4. Figure S1: Dendrogram showing fingerprinting profiles of K. pneumoniae phenotypically ESBL/AmpC and carbapenems producer strains isolated from Farms and supermarkets (vegetable, soil, and irrigation water samples). The profiles were obtained with ERIC‐PCR. Figure S2: The dendrogram fingerprinting profiles of E. asburiae , K. variicola, and K. oxytoca phenotypically ESBL/AmpC and carbapenems producer strains isolated from Farms and supermarkets (vegetable, soil, and irrigation water samples). The profiles were obtained with ERIC‐PCR. Figure S3: Dendrogram fingerprinting profiles of A. baumannii , A. pittii , K. aerogenes , and E. bugandensis phenotypically ESBL/AmpC and carbapenems producer strains isolated from Farms and markets (vegetable, soil, and irrigation water samples). The profiles were obtained with ERIC‐PCR. Figure S4: Dendrogram fingerprinting profiles of E. cloacae , E. kobei, and C. braakii phenotypically ESBL/AmpC and carbapenems producer strains isolated from Farms and supermarkets (vegetable and soil samples). The profiles were obtained with ERIC‐PCR. Figure S5: (A) Amplification of the donor and transconjugant ESBL and Carbapenemases encoded genes. M: “1 kb Thermo DNA ladder,” 1: Donor ETKP 42, 2: TXG ETKP 42, 3: Donor ETECO 160, 4: TXG ETECO 160, 5: Donor ETECO 169, 6: TXG ETECO 169, 7: Donor ETECO 169, 8: TXG ETECO 169, 9: Donor ETECO [file FSN3-14-e71761-s001.zip › FS5 (B).png]
